# Central retinal artery occlusion: a stroke of the eye

**DOI:** 10.1038/s41433-024-03029-w

**Published:** 2024-03-28

**Authors:** Celia Chen, Gurfarmaan Singh, Reema Madike, Sudha Cugati

**Affiliations:** 1 Department of Ophthalmology, Flinders Medical Centre and Flinders University, Adelaide, South Australia Australia; 2 The University of Adelaide School of Medicine, Adelaide, South Australia Australia; 3 Department of Ophthalmology, Modbury Hospital, Adelaide, South Australia Australia

**Keywords:** Optic nerve diseases, Eye manifestations

## Abstract

Central retinal artery occlusion (CRAO), like a stroke in the brain, is a critical eye condition that requiring urgent medical attention. Patients with CRAO present with acute loss of vision and the visual prognosis is poor with low chance of spontaneous visual recovery. Moreover, the risk of developing ischaemic heart disease and cerebral stroke is increased due to the presence of underlying atherosclerotic risk factors. Currently, there is no officially recommended treatment for CRAO. This review will describe the anatomy, pathophysiology, clinical features of CRAO, as well as exploring existing and potential future approaches for managing the condition.

## Abstract (zh)

视网膜中央动脉阻塞 (CRAO) 与脑卒中一样, 是一种需要紧急救治的重要眼病。CRAO患者表现为急性视力丧失, 视力预后较差, 自发视力恢复的机率较低。此外, 由于潜在动脉粥样硬化危险因素的存在, 增加了患缺血性心脏病和脑卒中的风险。目前, 还没有官方推荐的CRAO治疗方法。这篇综述将描述CRAO的解剖、病理生理学、临床特征, 并探索现有和潜在的未来治疗方法。

## Introduction

An arterial occlusion of the eye is the ocular equivalent of a cerebral stroke. The clinical presentation depends on the site and degree of occlusion and the visual outcome is dependent upon if the ischaemic changes are transient or permanent.

At presentation, a symptomatic retinal artery occlusion (RAO) is an ophthalmic emergency. Patients typically present with sudden, painless, monocular vision loss and the visual prognosis can be poor with 61% of central retinal artery occlusion (CRAO) patients having vision of counting finger or worse. At present, there is no definitive evidenced based treatment guideline for RAOs that has been found to be superior in terms of efficacy for the condition [1].

In 2013, the American Heart Association (AHA)/American Stroke Association updated their stroke classifications and included CRAO and branch retinal artery occlusion (BRAO) [2]. The aetiology and pathophysiological mechanisms of ischaemic stroke and RAO are very similar with thromboembolism resulting in vascular occlusion and end organ damage. There is an analogy in the management principle; the management of acute CRAO is like the principles of stroke treatment, with an aim to restore perfusion if possible and to optimise atherosclerotic control to prevent further ischaemic events.

This review aims to provide an overview of RAO, an update on its management and a practical guideline to manage patients presenting with RAOs.

## Methods

A structured search was conducted using the MEDLINE database (via PubMed) for this review. The following terms were used in various combinations: ‘central retinal artery occlusion,’ ‘branch retinal artery occlusion,’ ‘retinal artery occlusion’ with ‘epidemiology,’ ‘pathogenesis,’ ‘classification,’ ‘investigation,’ ‘management’ and ‘therapeutics,’ to identify relevant articles for inclusion in this review. All articles’ titles and abstracts were used to select articles, if deemed to be appropriate for inclusion, the full article was downloaded and analysed by the authors. There was no restriction in study design or article type for inclusion in this review, all bibliographies from included studies were reviewed to identify any additional relevant articles. All studies published until the 8th of November 2023 were assessed for inclusion.

## Epidemiology

CRAO is relatively rare; earlier literature estimated its incidence to be one patient affected per 100,000 in a calendar year [3]. In the United States of America, the age- and sex-adjusted incidence of CRAO is 1.9/100,000 person-years [4]. Interestingly, the crude incidence rate of CRAO in Japan was 5.84/100,000 person-years; a significant proportion of this could be attributed to the Japanese population being a super-aged society compared to the global population suggesting the incidence increase with age [5].

BRAO is another subtype of RAO, and the incidence is slightly higher at 4.99/100,000 person-years [6]. This more recent population data from around the globe suggests the incidence of RAOs is higher than initially estimated. Furthermore, the global population overall is ageing, with the average life expectancy significantly increasing year on year. This, in combination with RAOs having the highest incidence in individuals over 80 years of age, creates the possibility of the frequency of RAO presentations increasing globally [7]. These trends form the basis for the increase in trials searching for a robust treatment for RAOs over the past several years.

## Anatomy and pathophysiology

**Fig. 1:**
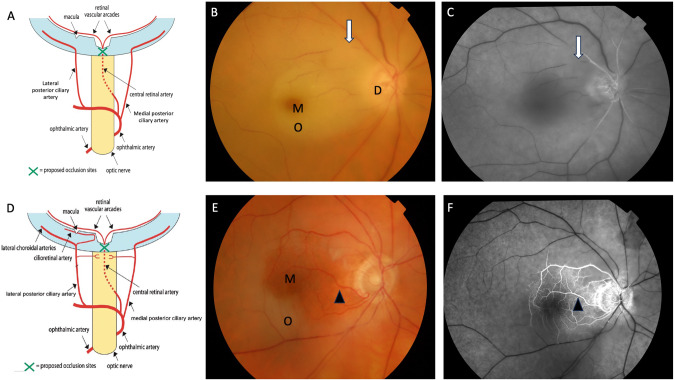
Vascular supply to the eye and clinical features in CRAO. **A** The central retinal artery is a branch of the ophthalmic artery. In central retinal artery occlusion (green cross marks site of occlusion), the blood supply to the retina is interrupted. **B** Clinical fundus photograph of the right eye in a patient with acute CRAO showing disc oedema (D), retinal oedema (O) around the macula (M) result in a cherry-red appearance of the macula and arterial attenuation (white arrow). **C** FFA at 58 s showing delayed arterial perfusion (white arrow) **D** Patients with a cilioretinal artery have supplied to the macula stemming from the short posterior ciliary artery. Therefore, in CRAO (green cross), the macula is supplied. **E** Coloured fundus photograph, and **F** FFA showing the perfused macula (M) supplied by the cilioretinal artery (arrowhead) but there is oedema around the macula (O). CRAO central retinal artery occlusion. FFA fundus fluorescein angiography.

RAO occurs secondary to occlusion of the central retinal artery (CRA) or the occlusion of a more terminal branch more distally [8]. The ophthalmic artery (OA) branches from the internal carotid artery and this is the origin of the CRA [9]. The CRA starts medial to the ciliary ganglion, extending into the orbital cavity. It then penetrates the dura mater of the optic nerve ~1 cm from the eye (Fig. 1A) [10]. The CRA continues to the optic nerve surface, following which it divides into superior and inferior branches. Each respective branch further subdivides into nasal and temporal tributaries. This vascular pathway is responsible for supplying the optic nerve surface and the inner retina of the eye. The ciliary arteries also originate from the OA, these vessels form the arterial blood supply of the choroid [11]. A disruption in either of these blood supplies, results in ischaemia and (if prolonged) necrosis of the retina.

A cilioretinal artery is present in 5–30% of patients [12]. The cilioretinal artery stems from the short posterior ciliary artery and supplies the macula (Fig. 1D). CRAO with cilioretinal sparing therefore preserves central vision due to perfused macula (Fig. 1E, F).

The most implicated pathologic aetiologies are thrombo-embolic secondary to atherosclerotic disease and platelet forming thrombi. The occlusion lodge at the narrowest point of the CRA; where it pierces the dura of the optic nerve and a common site of thrombotic occlusion is near the lamina cribosa [13]. A recent study noted ~55% of CRAOs had an identifiable embolic source [14]. The majority of emboli arise from the carotid artery and are less likely to be of valvular aetiology [15]. Animal models suggest if the arterial occlusion can be cleared within 97 min, then it is possible to completely reverse retinal ischaemia and thus recover visual acuity [16]. Partial recovery are observed up to 240 min from the time of occlusion.

**Fig. 2:**
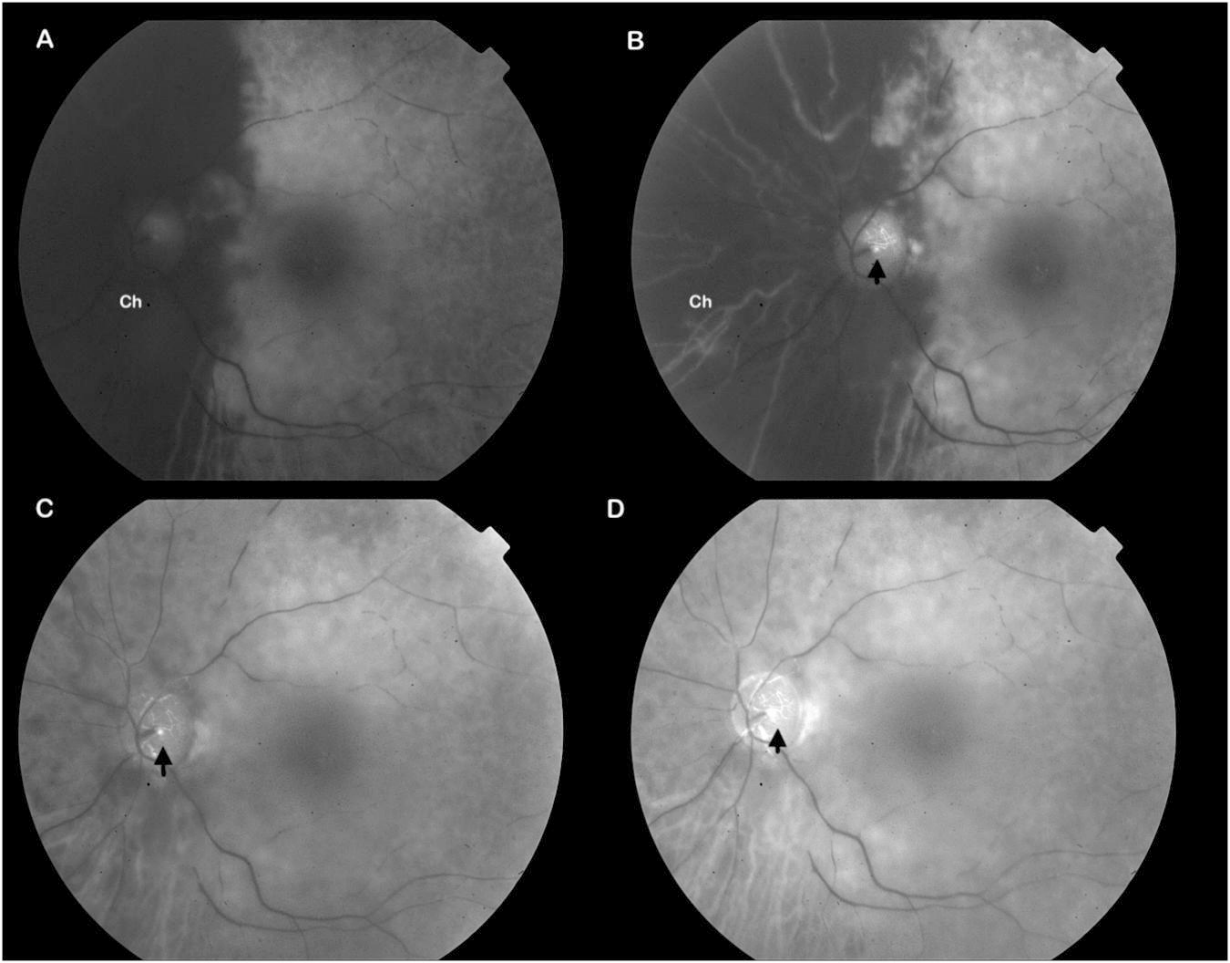
Fundus fluorescein angiography (FFA) image of arteritic L CRAO. **A**, at 22 s, **B**, 38 s, **C** 1 min 42 s and **D**, 5 min 10 s, showing vasodilation but non-filling of the retinal arteries at the level of the disc (arrow) as well as significant choroidal ischaemia (Ch).

Arteritic CRAO accounts for only a minority of CRAO presentations and is often a consequence of an inflammatory pathology; it is a large vessel ischaemia resulting in both changes at the level of the retina and choroid (Fig. 2). The most commonly associated condition is giant cell arteritis (GCA), however conditions such as Susac syndrome, systemic lupus erythematosus and granulomatosis with polyangiitis have also been associated [17–19]. In patients with GCA, there is intimal hyperplasia occluding the lumen, this results in subsequent ischaemia [1].

## Clinical features

In CRAO and related syndromes, patients typically present with sudden onset pain, unilateral and severe vision loss. The patients present with poor vision, an acuity of counting fingers or worse and the loss of vision can be within minutes [3, 13, 20]. Unfortunately, the presentation of CRAO patients to healthcare practitioners is significantly more delayed, particularly when compared to ischaemic cerebral stroke and acute coronary syndromes. Only 32% of CRAO patients presented directly to an ophthalmologist following onset of symptoms, with the mean time of symptom onset to review by any medical practitioner being 31.2 h [21]. The overwhelming majority of patients initially present to their general practitioner. The mean delay from the referring source to assessment by an in-hospital ophthalmologist was 5.2 h (median 3.3 h, range 50 min to 24 h). This delay was, on average, shorter for patients referred directly to an ophthalmology clinic. A more recent study suggests there may be greater awareness of the urgency to get treatment for acute monocular vision loss amongst patients. Shah et al. [22] found 51% of patients contacted the healthcare system within 4.5 h of symptom onset, however the initial review being by an ophthalmologist remained similar at 27.5% of presentations. This highlights the importance of raising awareness for the time sensitive nature of RAO at the level of the patient, the general practitioner, and the requirement for a prompt referral pathway for these patients with acute monocular vision loss to an ophthalmologist.

Risk factors for RAOs are similar to ischaemic cerebral stroke and myocardial infarctions, and include: advanced age (over 60), male gender, cardiovascular disease and smoking [15]. A significant proportion of CRAO patients have undiagnosed cardiovascular disease risk factors at time of presentation. Callizo et al. [23] found 78% of CRAO patients had at least one newly identified risk factor after diagnosis. Furthermore, CRAO maybe a precursor for stroke, with 13% of patients included in this study having a subsequent stroke; with just under half experiencing this within 4 weeks of CRAO onset.

In the setting of an acute event, the following findings are most commonly seen on fundus examination: a cherry-red spot (90%), retinal opacity in the posterior pole (58%), disc pallor (39%), retinal artery attenuation (32%), disc oedema (22%) and box-carring (19%) (Fig. 1B, C) [24]. At the advanced stage of disease, optic atrophy, retinal artery attenuation and cilioretinal collaterals were the three most frequently noted fundus findings [24]. Intra-arterial emboli are seen in around 20% of fundi examinations; the three most frequent emboli are cholesterol, calcium and platelet-fibrin [25]. Visual field defects in patients suffering a CRAO usually result in a central/centrocaecal scotomas (19%) and temporal island (59%) defects [26]. Those with a cilioretinal sparing CRAO may have preserved central area but peripheral visual field loss. Positive prognostic indicators of visual field improvement are mild CRAO stages, a good baseline visual acuity, mild retinal changes and less severe visual field defects at time of presentation.

**Fig. 3:**
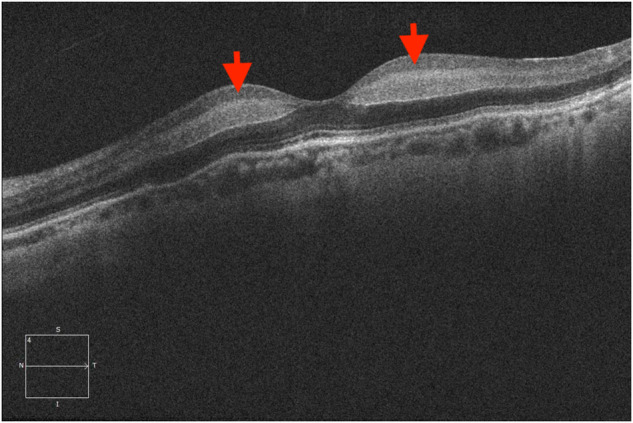
Paracentral acute middle maculopathy in a case central retinal artery occlusion showing band-like hyperreflective focal or diffuse lesions visible at the level of the INL corresponding to superficial INL ischaemia on OCT angiography.

Paracentral acute middle maculopathy (PAMM) has been defined with the presence of a hyperreflective parafoveal band at the level of the inner nuclear layer (INL) on spectral-domain optical coherence tomography (SD-OCT) (Fig. 3). PAMM is commonly associated with CRAO [27]. This clinical entity has been shown to be associated with several retinal vascular diseases is postulated to be due to localised ischaemia of the intermediate plexus secondary to an underlying ischaemic insult such as CRAO [28].

## Classification

**Fig. 4:**
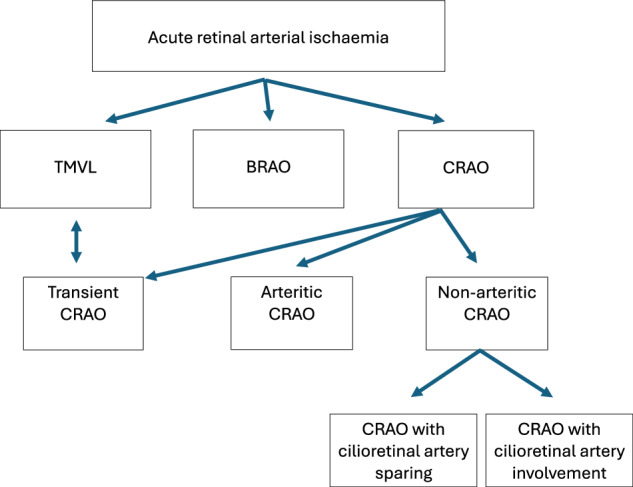
Classification of acute retinal arterial ischaemia. TMVL transient monocular vision loss. BRAO branch retinal artery occlusion. CRAO central retinal artery occlusion.

The classification of acute retinal arterial ischaemia is divided into transient monocular vision loss (TMVL), BRAO and CRAO (Fig. 4). [29].

CRAO can further be classified into the entities: transient non-arteritic CRAO, arteritic CRAO, non-arteritic CRAO. The non-arteritic CRAO can be associated with or without cilioretinal artery sparing [1, 8]. The current above classification is based on the anatomy and pathophysiology.

Schmidt et al. [30] classified the CRAO as incomplete, subtotal and total based on the clinical criteria such as vision loss and extent of retinal oedema as well as a delay in arterial blood flow on Fundus fluorescein angiography (FFA). However, the classification is subjective and does not provide quantitative information. The description of CRAO with PAMM has generated interest to use SD-OCT reflectivity measurement as an estimate the grade of retinal ischaemia in RAO [31]. However, this is not yet standardised.

## Clinical workup

All patients presenting with suspected CRAO, and related stroke syndromes should undergo prompt, extensive ocular and systemic clinical workup. The history should include a precise timeline of the onset of ocular symptoms, any systemic vascular disease symptoms, past medical history of vascular conditions and smoking history. Examination with visual acuity, relative afferent pupillary defect, colour vision and visual field testing assess the degree of the optic nerve function. The optic nerve and retinal examination may be supported with auxiliary ophthalmology investigations to visualise the optic and retina structure. These may be in the form of coloured fundus photograph, optical coherence tomography (OCT) and/or FFA.

OCT and OCT angiography (OCT-A) can be invaluable tools to evaluate degree of retinal ischaemia, disease stage and prognostication of disease. Mild CRAO is usually noted to have middle retinal layer opacification initially (PAMM), with eventual progression to inner retinal layer thinning over time. In moderate CRAO, there is total inner retinal layer opacification and similar progression to inner retinal layer thinning being seen. OCT findings in severe CRAO are more variable, with patients being observed to have intra-retinal fluid, neurosensory detachment, internal limiting membrane detachment, hyperreflective foci and opacities in their posterior vitreous [32]. OCT-A is a relatively new non-invasive imaging modality; this technology is seeing increasing incorporation into clinical practice when managing diseases affecting retinal vasculature. In the case of CRAO, OCT-A imaging demonstrates a reduction in the flow velocity through the superficial and deep capillary plexuses [33]. Interestingly, the overall thickness of the 3 × 3 mm imaged cube, parafoveal and central macular thicknesses are all increased in RAO eyes compared to non-RAO counterparts. Similarly, if a diagnosis of a RAO is inconclusive, FFA can be a useful investigation. CRAO findings can include delayed arterial perfusion, leakage at the disc and/or vessel walls (Fig. 1C) [33].

Patients with CRAO are at significant risk of future cardiovascular and cerebrovascular events. They often have undiagnosed risk factors that may be modifiable for secondary vascular prevention [34]. The NASCET study [35] showed that the overall vascular risk (including myocardial ischaemia and cardiovascular death) is as high for patients with a retinal ischaemia as it is for those with a cerebral TIA, emphasising the need for TIA prevention. There are several evidenced based recommended guidelines for workup of these patients for TIA prevention such as the NICE guidelines for TIA [36] and American Academy of Neurology TIA guidelines [1]. A collation of the recommended tests based on the guidelines is included Table 1. Workup should include vascular imaging and bloods for cardiovascular disease risk factors (e.g., lipid studies, HbA1c). Neuroimaging should also be given consideration and 30% of acute CRAO and 25% of BRAO patients have associated cerebral ischaemic changes on medical resonance imaging [1, 34, 35]. In patients suspected to have atrial fibrillation, Holter monitoring and echocardiography may also be of benefit. Less commonly, RAOs can occur in individuals under the age of 50 with no vascular risk factors. In these patients, a more extensive investigation panel including a hypercoagulable screen (protein C&S, factor V Leiden, anti-phospholipid antibody), vasculitis screen (ANA, ENA, ANCA, ACE) and blood film (myeloproliferative or sickle cell disease) should also be ordered [13].

**Table 1:** Suggested history and investigations for patients with CRAO.

Arteritic Cause	Investigation
History to investigate common risk factors	Family history of cardiovascular and cerebrovascular diseases Diabetes mellitus Dyslipidaemia Valvular heart disease Smoking Past medical history of TIAs or angina
Blood investigations for common vascular risk factors	Blood pressure Fasting blood glucose level Fasting cholesterol levels
Investigations to identify if it is from an embolic source	Duplex carotid ultrasound Echocardiogram
Investigations for young patients (<50 years) with no vascular risk factors	Hypercoagulable screen (protein C&S, factor V Leiden, anti-phospholipid antibody) Vasculitic screen (ANA, ENA, ANCA, ACE) Myeloproliferative or sickle cell disease (blood film)

## Management

The management of CRAO and related syndromes in recent times has become an increasing area of interest. This is due to the combination of significant visual morbidity associated with the condition, and drawing synergy from stroke management to look at acute reperfusion strategies. The American Heart Association Scientific Statement [1] on the management of CRAO has noted considerable variability in management patterns among practitioners, institutions and subspecialty groups. The development of local networks require collaboration among optometrists or general practitioners, ophthalmologists and stroke neurologists to facilitate such evaluations. This may be in a rapid-access transient ischaemic attack clinic, in an emergency department-observation unit, or with hospitalisation, depending on local resources. The role of the treating ophthalmologist is to diagnose the acute CRAO and to be aware of the local resources available to the patient.

In general, the management of RAO is divided into three phases:

Acute: to determine if reperfusion of the occluded retinal artery is possible

Subacute: to prevent local complications

Chronic: secondary prevention to optimise atherosclerotic control and prevent future events

### Acute treatment options

Time is critical in acute CRAO. Duration of ischaemia is directly associated with ganglion cell loss, with 240 min being the cut-off for massive irreversible retinal ischaemia [37]. This is similar to the current thrombolysis protocol in stroke to deploy clot busting agent if a stroke presents within a 4.5 h window.

Thrombolysis is to use a clot busting agent to dissolve an arterial clot and restore perfusion. Indications for thrombolysis include ischaemic cerebral stroke presenting within 4.5 h of onset, acute coronary syndrome with >2 h delay to angioplasty and large pulmonary embolisms with haemodynamic instability [38, 39]. In the case of CRAO and related syndromes, intravenous and intra-arterial thrombolysis have both been investigated. The initial thrombolytic agents include urokinase and streptokinase, but more recent thrombolytic agents include tissue plasminogen activator (tPA), alteplase and tenecteplase. Tenecteplase is of current research interest. It is genetically modified version of alteplase with higher fibrin specificity, increased resistance to plasminogen activator inhibitor-1 inactivation and longer half-life allowing for a quick bolus delivery. However, it is FDA approved for acute myocardial infarction but not in acute ischaemic stroke. Tenectaplase is being increasingly used off label due to the advantages of a single-dose intravenous administration and also being more cost effective compared to alteplase [40].

The thrombolytic agent used depend on the stroke thromobolysis protocol for each institution. Intravenous thrombolysis in CRAO utilises the stroke thrombolysis protocol and [41] the current treatment window for thrombolysis in stroke is less than 4.5 h of treatment onset [42, 43]. However, there is no standardised protocol or recommendation of time window in CRAO. Interventional case series have reported a visual acuity improvement between ~30 and 55% of patients who received IV-tPA [44–46]. In the first RCT on intravenous tPA in CRAO, a 24-h window was employed to maximise recruitment. The patients were stratified based on time window between 0–6, 6–12 and 12–24 h in the protocol. The study found that the only people who improved three of more lines of visual acuity were those who received thrombolysis within 6 h [47]. However, the improvement was not sustained at their 6 months follow-up. This was attributed to possible re-occlusion of the CRA, with the authors suggesting that adjuvant anticoagulation should be considered [47].

There are presently three major trials expected to be completed in the next 2 years which hold the potential to confirm the definitive efficacy of IV-tPA in CRAO and provide an evidence-based treatment. The first of the RCTs is THEIA (A Phase III Randomized, Blind, Double Dummy, Multicentred Study Assessing the Efficacy and Safety of IV Thrombolysis (Alteplase) in Patients With acute Central retinal Artery Occlusion) [48]. This trial commenced mid 2018 and is expected for completion in January 2024, the study is aiming to recruit 70 participants and at present is not actively recruiting. The second trial underway in Norway is TenCRAOS (TENecteplase in Central Retinal Artery Occlusion Study), this study is actively recruiting with a goal to evaluate a total dataset of 78 patients. This trial has an expected completion date of early to mid 2024 [49]. The largest of the three major trials underway is REVISION (Early Reperfusion Therapy With Intravenous Alteplase for Recovery of VISION in Acute Central Retinal Artery Occlusion). The study is aiming to include 1400 patients within their cohort and is expected for completion in late 2025 [50]. Thrombolysis may be considered in CRAO, but not in BRAO. Successful thrombolysis in acute CRAO requires a collaboration of the ophthalmologist to make the prompt diagnosis within the correct time window, coupled with the expertise of a stroke thrombolysis set up. A report from a tertiary institution with CRAO thrombolysis protocol in place showed that only 3/181 CRAO patients who presented within the 4.5-h time window patients received IV thrombolysis, emphasising the difficulty in administering very acute treatments for CRAO [51]. The study identified the need for an accelerated diagnostic pathway protocol for patients with acute CRAO for the potential acute treatments.

Intra-arterial is another method to deliver tPA (IA-tPA) in CRAO and has two potential advantages over other methods of administration; it requires lower doses for therapeutic effect and may have a greater time to treatment window. A cohort study found patients who underwent IA-tPA therapy were 36 times more likely to have an improvement in their visual acuity compared to controls, they were also 13 times more likely to have an improvement of three or more lines on visual acuity testing [52]. However, the earliest RCT suggested no difference in visual acuity improvement when comparing intra-arterial thrombolysis to conservative standard treatment for CRAO of 24 h onset [53]. The major disadvantage is the requirement for interventional neuro-radiology expertise to give the intra-arterial thrombolysis delivery and the potential complications from the intervention, including transient ischaemic attacks and strokes [54]. A 37.1% of adverse event rate was reported in IA-tPA patients, significantly higher than standard conservative management [55]. Overall, a recent pooled meta-analysis supported the use of IA-tPA to conservative therapy, although significantly larger RCTs are required [55]. A key limitation of IA-tPA is the need for access to an interventional neuroradiologist. This is a speciality only often based at large metropolitan hospitals. Therefore, its application may be limited in diverse clinical settings.

Several other methods have been described in acute CRAO management (Table 2). These acute therapies, singly or in combination, do not alter the clinical outcome. Rudkin et al. [56] evaluated the acute treatment of CRAO at Johns Hopkins Hospital (JHH) in the United States versus Flinders Medical Centre (FMC) in Australia. More patients in the JHH cohort underwent paracentesis, ocular massage or were treated with intraocular hypotensive agents (76%) than in the FMC cohort (26%); however, there was no significant difference in visual outcome between the two cohorts (*p* = 0.114). This suggests a lack of efficacy of current standard treatment in acute CRAO. Some of the interventions may also bear high risk of complications. For instance, transluminal Nd: YAG laser therapy tries to lyse an embolus or cause its migration into the vitreous. The most common complication is haemorrhages, with vitreous haemorrhages accounting for 54% of adverse haemorrhages [57]. Some of the therapies like hyperbaric oxygen (HBOT) includes 100% oxygen inhalation to increase the amount of oxygen dissolved in body tissue [58]. In a recent study, HBOT was given at 90-min sessions three times in the first 24 h, followed by once daily until there was no further improvement noted in the vision after two consecutive treatments [59]. It was found that HBOT is very effective in CRAO, provided the macular is not ischaemic and has not developed cherry-red spots, with greatest improvement seen if patients are treated within 12 h of onset of symptoms [60]. A recent meta-analysis suggested that HBOT does not improve final visual outcomes and the risks associated include barotrauma, tympanic membrane rupture and generalised seizures due to oxygen toxicity to the central nervous system [61].

**Table 2:** Acute CRAO management strategies and options.

Treatment strategy	Treatments described
To increase mean perfusion pressure across the optic nerve head by reducing intraocular pressure	AC paracentesis Intravenous acetazolamide Pars plana vitrectomy
To vasolate the central retinal artery to increase perfusion	Inhaled carbogen Sublingual isosorbide dinitrate Hyperbaric oxygen Pentoxyphyllline
To dislodge the emboli	Ocular massage Transluminal Nd: YAG laser Pars plana vitrectomy

### Subacute management of CRAO – preventing secondary ocular complication

CRAO can cause ocular neovascularisation due to chronic retinal ischaemia resulting from reperfusion failure. Prompt treatment including pan retinal photocoagulation and neovascular glaucoma should be considered if the patient develops neovascularisation. A direct temporal relationship between CRAO and ocular neovascularisation has been reported, with a prevalence of 2.5–31.6% [62, 63]. The mean time for neovascularisation to be observed post CRAO was 8.5 weeks (range of 2–16 weeks) [62]. Therefore, neovascularization does occur in CRAO, similar to retinal vein occlusion. Regular ophthalmology review in the subacute stage is important up to 4 months after CRAO to prevent local complications.

### Secondary prevention to optimise atherosclerotic control and prevent future events

There is a higher risk of future ischaemic events in patients with CRAO [63]. A retrospective audit found that 64% of patients had at least one undiagnosed vascular risk factor, with 36% of these patients having hyperlipidaemia at the time of the CRAO event [64]. Therefore, secondary prevention should involve multidisciplinary collaboration with a neurologist, ophthalmologist and primary care physician. Ophthalmological follow-up is important to optimise the residual vision, monitor for neovascularisation-related complications and preserve the health of the contralateral eye [65]. The neurologist works in collaboration to determine the cause and initiate appropriate pharmacological secondary prevention. Once the cause is determined, it is important to work with the general practitioner to control systemic atherosclerotic risk factors, including hypertension, hyperlipidaemia, diabetes, obesity and obstructive sleep apnoea. Smoking cessation, diet and regular physical activity are also important for secondary prevention of CRAO [1, 66].

It is recommended to follow established professional guidelines as per the AHA for transient ischaemic attacks or minor strokes [1]. For patients who do not have an indication for anticoagulation or surgery, it is therefore reasonable for patients to receive antiplatelet therapy. In patients without contraindications, the guidelines recommend an initial 21-day course of dual antiplatelet therapy, followed by long-term treatment with a single antiplatelet agent, which is typically aspirin 81 mg daily or clopidogrel 75 mg daily.

## Recommended management

Based on currently available literature, patients presenting with CRAO, and related syndromes should be promptly reviewed with a view to initiate acute treatment if patient present within an allocated time window for consideration of thrombolysis. Patients should undergo thorough history taking and clinical assessment. Consideration should be given to any symptoms concerning for GCA, additional neurological symptoms and contraindications for tPA therapy. A review by an ophthalmologist should also be performed upon presentation. If patients are within the 4.5-h treatment window, they may be considered for IV-tPA with consultation from the stroke unit.

From an institutional perspective, the AHA recommend modification of current hospital code stroke protocols for CRAOs and related syndromes. They recommended the addition of a funduscopic examination and screening for arteritis [1]. The guidelines emphasised the importance of stroke centres developing relationships with community optometrists and ophthalmologists to promote efficient pathways for RAO patients. Grory et al. also suggested public health campaigns to include CRAO symptoms as a component of potential stroke features to improve awareness regarding the time sensitive nature of RAOs [1].

In cases where the patient has a delayed presentation and is ineligible for IV-tPA, patients can commence antiplatelet therapy and be referred to stroke clinic for systemic workup with a view for secondary prevention.

## Future directions

There is a need for Level II evidence double-blinded randomised controlled trial to address the efficacy of thrombolysis compared to placebo in treating early CRAO within suitable therapeutic treatment window. The exploration of novel thrombolytic agent such as tenecteplase holds promise for forthcoming research endeavours. A collaborative approach is important to develop a system of care for the urgent recognition, triage, and management of CRAO in a manner like cerebral ischaemic stroke.

## Conclusion

CRAO is an ocular emergency and is analogous to an ischaemic stroke. The risk factors associated with non-arteritic CRAO is like that of cerebrovascular accident or myocardial infarction. Whilst various reports suggest different treatment modes for CRAO, there is little evidence supporting an optimal management plan. After taking a thorough history, ocular examination and investigations, the principles of management of CRAO includes acute management to restore blood flow, subacute management to prevent secondary complications and secondary prevention.
